# A case of squamous cell carcinoma of the breast achieved a pathological complete response after dose-dense AC + dose-dense PTX

**DOI:** 10.1186/s40792-023-01719-3

**Published:** 2023-08-02

**Authors:** Misato Araki, Koshi Matsui, Kohji Takagi, Emi Kanaya, Shinichi Sekine, Shiho Nagasawa, Toru Watanabe, Takeshi Miwa, Katsuhisa Hirano, Takamichi Igarashi, Haruyoshi Tanaka, Kazuto Shibuya, Isaya Hashimoto, Shozo Hojo, Isaku Yoshioka, Tomoyuki Okumura, Kenichi Hirabayashi, Tsutomu Fujii

**Affiliations:** 1 Department of Surgery and Science, Faculty of Medicine, Academic Assembly, University of Toyama, 2630, Sugitani, Toyama, 930-0194 Japan; 2 Department of Diagnostic Pathology, Faculty of Medicine, Academic Assembly, University of Toyama, 2630, Sugitani, Toyama, 930-0194 Japan

**Keywords:** Squamous cell carcinoma of the breast, Pathological complete response, Neoadjuvant chemotherapy, Breast-conserving surgery

## Abstract

**Background:**

Squamous cell carcinoma (SCC) of the breast is a rare form of breast cancer, accounting for approximately 0.1% of all breast cancers. It is known for its rapid tumor growth and poor prognosis with no established treatment.

**Case presentation:**

A 56-year-old woman was diagnosed with breast SCC with axillary, supraclavicular and internal thoracic lymph node metastases. She received neoadjuvant chemotherapy (NAC) with dose-dense doxorubicin and cyclophosphamide (AC) followed by dose-dense paclitaxel (PTX). This treatment resulted in a pathological complete response (pCR) after breast-conserving surgery. The patient was then treated with radiotherapy. She remained free of recurrence for three years postoperatively.

**Conclusions:**

We report a rare case of breast SCC treated with preoperative dose-dense chemotherapy, resulting in pCR and allowing breast-conserving surgery.

## Background

Squamous cell carcinoma (SCC) of the breast, classified as a metaplastic carcinoma by the World Health Organization (WHO) classification, is a rare form of breast cancer accounting for approximately 0.1% of all breast carcinomas [1].

It is characterized by rapid tumor growth and poor prognosis, with no established treatment. We present a case of squamous cell carcinoma treated with dose-dense doxorubicin and cyclophosphamide (AC) followed by dose-dense paclitaxel (PTX), resulting in a pathological complete response (pCR) after breast-conserving surgery.

## Case presentation

**Fig. 1:**
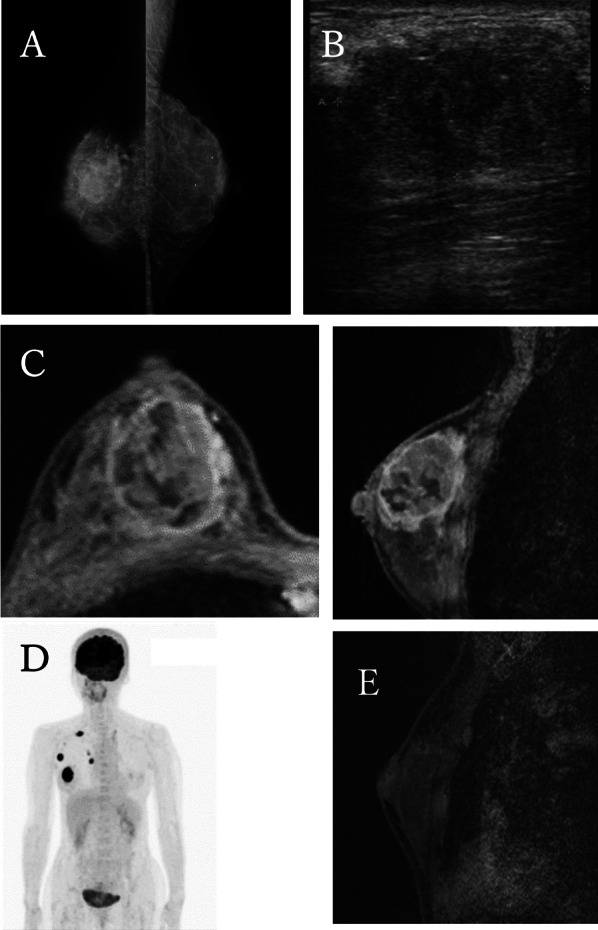
**A** Mammography revealed a hyperintense mass in the right breast. **B** Ultrasound showed a 35 mm irregular mass. **C** Magnetic resonance imaging (MRI) before NAC showed a massive lesion in the right area. The lesion was ring-shaped, contrast-enhancing, with a low-contrast zone. **D** Fluorodeoxyglucose positron emission tomography (FDG-PET) detected advanced accumulation in the right breast mass, axillary lymph nodes, right supraclavicular lymph nodes and right internal thoracic lymph nodes. **E** MRI after NAC showed a massive lesion was significantly reduced

A 56-year-old female with no significant medical or family history presented to our hospital with the chief complaint of a mass in the right breast. The Mammography, breast ultrasonography, CT, MRI and FDG-PET scans revealed a 35 mm mass in the right breast (Fig. 1). Core needle biopsy identified squamous cell carcinoma of the breast. The FDG-PET scan showed metastases in the axillary, supraclavicular and internal thoracic lymph nodes, giving a clinical stage of cT2N3M0, stage IIIC (Fig. 1).

**Fig. 2:**
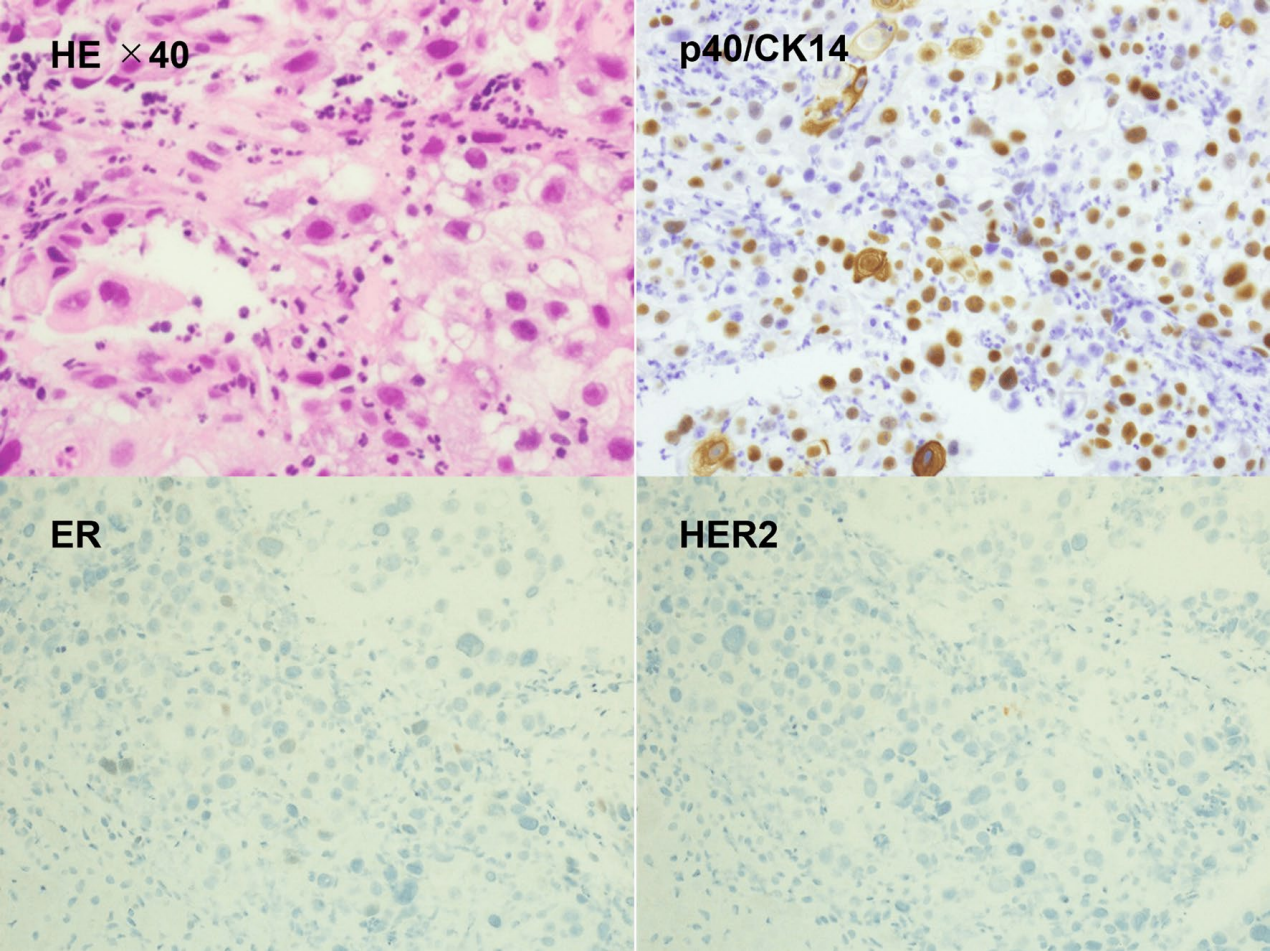
Core needle biopsy specimen of the tumor. The histological findings were focally positive for p40/CK14 staining, negative for hormone receptors and negative for HER2. The diagnosis was triple negative squamous cell carcinoma

Immunohistochemistry showed the tumor to be hormone receptor-negative, HER2-negative with a Ki-67 of 90% and triple negative (TN) breast cancer (Fig. 2).

The patient received 4 cycles of AC (doxorubicin 60 mg/m^2^ and cyclophosphamide 600 mg/m^2^) and 4 cycles of PTX (paclitaxel 175 mg/m^2^) with pegfilgrastim 3.6 mg as NAC. There were no serious adverse events. After NAC, the tumor was significantly reduced and showed ycT1N0M0 ycStage I on MRI (Fig. 1E). The patient underwent breast-conserving surgery and sentinel node biopsy, and postoperative histopathology showed no cancer cells.

After surgery, the patient received 50 Gy/25 fractions radiation to the residual breast and supraclavicular and internal thoracic lymph nodes and has remained free of recurrence for 3 years postoperatively.

## Discussion

SCC of the breast, classified as a metaplastic carcinoma in the WHO classification system, is a rare form of breast cancer, accounting for 0.06–0.1% of all cases [1]. As a result, there is no established treatment strategy, and treatment is often determined by clinical features [2]. There have been several reports of SCC cases achieving pCR by NAC (Table 1). A significant number of these cases were treated with anthracycline/taxane therapy. These cases reported in Table 1 might be very limited cases because SCC breast cancer is too aggressive subtypes to be cured [2]. Also, there have been reports of poor efficacy of anthracycline in breast SCC [2]. On the other hand, the Japanese guideline recommends a dose-dense anthracycline/taxane regimen for the treatment of TN breast cancer [9]. The Norton-Simon hypothesis states that sensitivity to chemotherapy is increased when tumor volume is small. Dose-dense therapy shortens the repopulation period so that the next dose is given when tumor volume is small. This allows administration at a time when the tumor is more sensitive to chemotherapy and a greater anti-tumor effect can be expected [9]. Therefore, we chose a dose-dense anthracycline/taxane regimen. However, platinum-based regimens are also recommended for treating TN breast cancer. In neoadjuvant chemotherapy trials, platinum-based regimens prolonged OS and improved pCR rates in TN breast cancer patients [10–12]. This demonstrates the benefit of platinum-based drugs for treating TN breast cancer. Although the efficacy of platinum-based regimens would be expected in this case, the lack of insurance coverage in Japan makes their use difficult. Recently, the use of the platinum-based KEYNOTE-522 regimen is now a promising option [13]. The KEYNOTE-522 trial of pembrolizumab for NAC in TN breast cancer showed improved event-free survival and pCR rate compared with the standard treatment [13]. The use of immune-checkpoint inhibitors for the treatment of TN breast cancer, including special types, is likely to increase in the future. KEYNOTE-826 study also demonstrated the efficacy of pembrolizumab in advanced recurrent cervical cancer, including squamous cell carcinoma [14]. These results suggest that immune-checkpoint inhibitors may be considered for use in SCC of TN breast cancer. Further case series are expected in the future.

**Table 1:** Cases of breast squamous cell carcinoma with pathological complete response

Case	Authors	Age	Pathological features	NAC regimens
1	Chen et al. [3]	48	ER(−), PR(−), HER2(−)Ki-67(60%)	TC 4 cycles
2	Alan et al. [4]	72	ER(≤ 1%), PR(−), HER2(−), Ki-67 (N/A)	wPTX + EC
3	Noro et al. [5]	40	ER(−), PR(−), HER2(−)Ki-67(N/A)	AC 1cycle + wPTX 12cycles
4	Usui et al. [6]	48	ER(−), PR(−), HER2(+)Ki-67(N/A)	Dose-dense AC 4cycles, HP + DTX 4cycles
5	Sakuma et al. [7]	57	ER(−), PR(−), HER2(−)Ki-67(N/A)	EC 4cycles DTX 4cycles
6	Dejager et al. [8]	61	ER(−), PR(−), HER2(+)Ki-67(N/A)	CDDP + 5-FU
7	Present case	56	ER(−), PR(−), HER2(−)Ki-67(90%)	Dose-dense AC 4cycles, Dose-dense PTX 4cycles

ER, estrogen receptor; PR, progesterone receptor; HER2, human epidermal growth factor receptor 2; N/A, not applicable; TC, docetaxel and carboplatin; wPTX, weekly paclitaxel; EC, epirubicin and cyclophosphamide; AC, doxorubicin and cyclophosphamide; HP, trastuzumab and pertuzumab; DTX, docetaxel; CDDP + 5FU, cisplatinum + 5-fluorouracil

In this case, at least three sentinel lymph nodes were identified, including the previously diagnosed positive node prior to NAC, all of which were found to be negative for metastases; therefore, axillary dissection was not performed. Lymph node metastasis is an established poor prognostic factor in SCC of the breast, and axillary dissection has been commonly performed in previously reported cases [15]. However, in cases where a pCR was achieved in the primary tumor, all dissected axillary lymph nodes were found to be negative for metastases, resulting in a favorable prognosis. The incidence of lymph node metastasis has also been reported to be low in upfront surgery cases [16].

These findings suggest that sentinel lymph node biopsy or tailored axillary surgery may be appropriate for cases of SCC in which NAC has resulted in a significant reduction in the size of the primary tumor.

Regarding the surgical approach after NAC, mastectomy is often performed when the primary tumor is large. Total mastectomy was performed in all cases of breast SCC with pCR after NAC (Table 1), and no partial mastectomy was performed. If NAC leads to tumor shrinkage, Japanese guidelines also recommend breast-conserving therapy [3]. In this case, partial mastectomy was performed due to the significant reduction achieved with NAC.

There is also evidence that postoperative radiotherapy (PORT) significantly improves overall survival in SCC of the breast, regardless of surgical technique [16]. In the current case, radiotherapy was administered to the supraclavicular and internal thoracic lymph nodes that were preoperatively positive for metastases, in addition to the breast that was preserved postoperatively. The patient progressed without local recurrence. If a prominent reduction is obtained in NAC, partial mastectomy and PORT might be considered in breast SCC.

## Conclusion

We encountered a case of squamous cell carcinoma of the breast where preoperative dose-dense chemotherapy was administered and breast-conserving surgery was possible. It was suggested that dose-dense therapy might be effective as NAC for breast SCC.

## Data Availability

The datasets of this case report are available from the corresponding author upon reasonable request.

## Abbreviations

SCC: Squamous cell carcinoma
NAC: Neoadjuvant chemotherapy
pCR: Pathological complete response
AC: Doxorubicin and cyclophosphamide
PTX: Paclitaxel
MRI: Magnetic resonance imaging
FDG-PET: Fluorodeoxyglucose positron emission tomography
TC: Docetaxel and carboplatin
EC: Epirubicin and cyclophosphamide
wPTX: Weekly paclitaxel
HP: Trastuzumab and pertuzumab
DTX: Docetaxel
CDDP + 5FU: Cisplatinum + 5-fluorouracil
